# The effects of pregnancy on the progression of maternal glomerular disease

**DOI:** 10.1590/2175-8239-JBN-2024-0205en

**Published:** 2025-08-01

**Authors:** Luiz Paulo José Marques, Lívia Menezes Salla, Lilimar da Silveira Rioja, Regina Rocco, Eugênio Pacelle Queiroz Madeira, Lygia Maria Soares Fernandes Vieira

**Affiliations:** 1 Universidade Federal do Estado do Rio de Janeiro, Hospital Universitário Gaffrée e Guinle, Departamento de Medicina, Rio de Janeiro, RJ, Brazil.

**Keywords:** Renal Insufficiency, Chronic, Glomerular Diseases, Pregnancy, Chronic Hypertension, Proteinuria, Pre-Eclampsia

## Abstract

**Introduction:**

Although most women with underlying glomerular diseases (GD) are of childbearing age, there is limited information on how pregnancy affects these conditions and maternal outcomes.

**Methods:**

We carried out a single-center retrospective cohort study involving 44 planned pregnancies in 38 patients with biopsy-proven GD. Patients were divided into three groups based on their pre-conception CKD-KDIGO classification: I) Stage 1–2: 27 pregnancies, II) Stage 3a–3b: 11 pregnancies, and III) Stage 4–5: 6 pregnancies. Clinical data included age, chronic hypertension (CH), serum creatinine, preeclampsia (PE), and proteinuria. We considered CH, CKD stage before pregnancy, and PE and nephrotic proteinuria (NPu) during pregnancy as risk factors for maternal GD progression.

**Results:**

We found that 8 women progressed to ESRD and began hemodialysis during pregnancy: 2 (7.8%) in Group I, 1 (9.0%) in Group II, and 5 (83.3%) in Group III. In the remaining 36 pregnancies, we observed a significant GFR loss (p < 0.0001) one year after pregnancy, and GFR loss was greater in group II than in I (p < 0.013). Low GFR rates before pregnancy and PE during pregnancy (p = 0.001) directly impacted GFR loss. We also observed a high incidence (63.6%) of adverse fetal outcomes.

**Conclusion:**

Although pregnancy is possible for women with GD, the impact of pregnancy in maternal GD continues after delivery. Having GD increases the risks of adverse pregnancy outcomes. The progression of GD is directly linked to the CKD stage before pregnancy and PE during pregnancy, and women in CKD stages 4–5 have a high risk of progressing to ESRD during gestation.

## INTRODUCTION

Chronic kidney disease (CKD) is a risk factor for adverse pregnancy outcomes, even in the early stages of the disease. CKD must be included among the significant risk factors such as diabetes and hypertension^1^. Studies have shown that 3 to 6% of women of childbearing age have CKD, and up to 3.3% of pregnant women have laboratory evidence of CKD^2,3^. The effect of pregnancy on renal disease and the impact of renal disease on pregnancy may be harmful.

Although most women with underlying glomerular diseases (GD) are of childbearing age, these diseases can be missed during pregnancy and only diagnosed later in life. An Australian study that diagnosed 0.3% of pregnant women with kidney diseases, immunologic diseases or glomerular diseases was only identified in 0.01%^4^.

Pregnancy brings about important changes in immunity and renal physiology, which can hasten GD progression. These changes include decreased kidney vascular resistance, associated with elevations in cardiac output and renal blood flow with a simultaneous 38–56% increase in glomerular filtration rate (GFR)^5^. This results in a physiological rise in protein excretion due to increased GFR and glomerular basement membrane permeability^6^. Another major adaptation is the modulation of innate and adaptive immunity to establish maternal tolerance to the semi-allogeneic fetus that expresses maternal and paternal antigens^7^.

Over the last few decades, there has been growing attention to the complex interactions between CKD and pregnancy, as well as the various aspects of pregnancy in women with kidney diseases. However, little is known about specific glomerular diseases other than lupus and IgA nephropathy, which occur more frequently^8,9^.

GD commonly affect women of reproductive age and can pose many challenges during pregnancy, including immunologic diseases, hypertension, proteinuria, and kidney tissue injury. However, there is limited information on the outcomes of such conditions, particularly in terms of maternal renal disease progression. It is essential to increase this information to enhance personalized and comprehensive preconception counseling, improve maternal-fetal outcomes, and customize clinical practice.

Therefore, we aimed to assess the impact of pregnancy on the progression of GD. We studied the effects of pregnancy on renal function, risk factors for adverse maternal outcomes, and maternal-fetal outcomes of these patients.

## METHODS

### Study Subjects

This study was carried out with pregnant women who had biopsy-confirmed GD before conception and received obstetric prenatal and nephrological care at Gaffrée and Guinle University Hospital from January 2009 to December 2019. All participants willingly provided written informed consent, and this clinical study was performed following the Declaration of Helsinki and approved by the local human research ethics committee with approval number: 3.046.179.

We excluded from the study diabetic nephropathy women due to peculiar maternal-fetal outcomes in diabetes^10^ and those who presented GD flare or de novo GD in the first year after giving birth.

All patients had planned pregnancy and underwent close monitoring by a specialized multi-professional team. A predefined laboratory and clinical evaluation protocol was followed at scheduled visits throughout the gestation^11^.

### Study Design

This descriptive, single-center retrospective cohort study was conducted with pregnant patients with primary or secondary GD, who had at least 12 months follow-up after delivery in our hospital. GFR was measured using the CKD-EPI equation before conception and 12 months after delivery. They were divided into three groups following CKD KDIGO classification before pregnancy: Group I (stage 1–2): GFR ≥ 60 mL/min/1.73m^2^, Group II (stage 3a–3b): GFR ≤ 59 and ≥ 30 mL/min/1.73m^2^ and Group III (stage 4–5): GFR ≤ 29 mL/min/1.73m^2^.

### Collection of Clinical Data

The following clinical data were collected: age, renal biopsy results, GD diagnosis time, arterial pressure, antihypertensive and immunosuppressive therapy use, and maternal-fetal outcomes.

Laboratory parameters recorded included: urea, serum creatinine (Scr) before conception and twelve months after the delivery, highest proteinuria (Pu) during pregnancy (urine protein levels were measured at the twelfth, twenty, thirty, and thirty-six pregnancy weeks or when necessary, through 24-hour urine collection), serum complement (C3 and C4), anti-DNAds and anti-PLA2R. The CKD-EPI equation was computed using the website https://qxmd.com, 2021 version, and ISN/RPS 2018 classification for lupus nephritis was applied.

### Maternal-Fetal Outcomes

Adverse fetal outcomes included perinatal mortality (PM, death of a viable fetus after 22 weeks of gestation or within 4 weeks of delivery, including stillbirth), preterm delivery (PD, delivery before 37 weeks of gestation), and low birth weight (LBW, baby weighing less than 2500 g at birth. Normal newborn (NN) is the term that refers to an infant who is born at full term and is stable and healthy.

Adverse maternal outcomes included preeclampsia (PE, defined as arterial hypertension (AH) accompanied by worsening Pu after 20 weeks of gestation and, for women with preexisting AH, PE was defined as worsening AH (increase in systolic blood pressure by 30 mm Hg and diastolic blood pressure by 15 mm Hg) associated with worsening Pu after 20 weeks of gestation), placental abruption (PA, occurs when the placenta partly or completely separates from the inner wall of the uterus before delivery), and progression to end-stage renal disease (ESRD, need for a regular course of long-term dialysis).

### Risk Factors to Maternal Glomerular Disease Progression in Pregnancy

Chronic hypertension (CH) and higher CKD KDIGO stages before conception, PE, and nephrotic proteinuria (NPu) during pregnancy were considered risk factors for maternal glomerular disease progression.

CH was defined as systolic blood pressure > 140 and diastolic blood pressure > 90, or patients on antihypertensive therapy before conception. NPu was defined as Pu ≥ 3.5 g/24 h. CKD KDIGO Stage: Chronic Kidney Disease KDIGO Classification Stage from 1 to 5.

### Statistical Analysis

The statistical analysis was done in GraphPad InStat-3 (GraphPad Software, San Diego, CA). Categorical and numerical data were expressed as means ± standard deviations; a T-test or analysis of variance (ANOVA) was used to compare data. Outcome data were compared using the Wilcoxon matched-pair test, and bivariate correlations of risk factors between groups were tested using Fisher’s exact test. Relative risk (RR) and 95% confidence intervals were calculated using standard methods. A P-value < 0.05 was considered statistically significant.

## RESULTS

This study included 38 women with mean age of 28.1 ± 4.7 (range: 18 to 39) years, who had long-lasting GD diagnosed from 18 to 172 (mean 62.3 ± 31.6) months through kidney biopsy before conceiving: 11 cases of focal segmental glomerulosclerosis (FSGS) with 12 pregnancies, 10 cases of lupus nephritis (LN) with 11 pregnancies, 6 cases of IgA nephropathy (IgAN), 3 cases of membranous nephropathy (MN) with 5 pregnancies, 2 cases of minimal change disease (MCD) with 4 pregnancies, 2 cases of membranoproliferative glomerulonephritis (MPGN), 2 cases of HIV associated nephropathy (HIVAN), 1 case of Alport syndrome (AS), and 1 case of reflux nephropathy (RN) associated to sclerosing nephropathy.

Patients were followed up for at least 12 months post-delivery. Of the 38 patients, 27 (71%) were from our Nephrology Unit, while 11 (29%) were referred from other hospitals during their first or second trimester of pregnancy. All pregnancies were planned, and teratogenic medications for hypertension, HIV, and immune suppression were replaced with safer alternatives therapy before conception and during GD flare or de novo GD.

The presence of NPu (3.5 ± 1.3, ranging from 0.7 to 8.2 g/24) were observed in 16 gestations, 8 of which showed GD flare or de novo GD signs with high levels of anti-PLA2R in 2 with MN, increased anti-DNAds associated with the decrease in serum complement in 4 with LN, and the remaining 2 were confirmed through renal biopsy during preg­nancy. However, proteinuria measurements were only obtained before conception in 16 of 44 pregnancies.

### Clinical Data of Pregnant Women

The women were divided into three groups according to CKD KDIGO classification. In Group I (Stage 1–2) there were 22 (57.9%) patients with 27 (61.3%) pregnancies aged 28.2 ± 4.3 (22 to 39) years and time of GD diagnosis of 61.1 ± 32.6 (range: 22 to 172) months (Table 1). In Group II (Stage 3a-3b) there were 10 (26.3%) patients with 11 (25%) pregnancies aged 29.7 ± 4.6 (23 to 38) years and time of GD diagnosis of 77.3 ± 27.6 (range: 47 to 122) months (Table 2). In Group III (Stage 4–5) there were 6 (15.8%) patients with 6 (13.7%) pregnancies aged 24.6 ± 5.6 (18 to 32) years and time of GD diagnosis of 47.0 ± 27.2 (range: 18 to 94) months (Table 3).

**Table 1:** Clinical data and maternal fetal outcomes of women in Group I

N	Diseases pre-Pregnancy	Age/Scr CKDEPI-Pre	Age/Scr CKDEPI-Post	Greater Pu in Pregnancy	Fetal Outcome	Maternal Outcome
1	LN IV	29/1.1	HDc	6.1	PM	ESRD
		69.8				
2	IgAN	22/0.9	HDc	4.2	PM	ESRD
		92.7				
3	LN IV/CH	26/1.2	27/1.4	3.6	PM	PE
		64.0	52.9			
4	FSGS/CH	31/1.0	33/1.2	3.9	PD/LBW	PE
		77.2	61.3			
5	IgAN/CH	30/0.9	32/1.0	4.6	PD/LBW	PE
		88.2	76.8			
6	IgAN/CH	22/1.0	23/1.2	2.9	PD/LBW	PE
		81.7	65.2			
7	LN II/CH	26/1.0	28/1.1	2.4	NN	
		79.7	70.2			
8	2nd Preg	29/1.0	30/1.1	1.0	NN	
		78.2	69.3			
9	FSGS/CH	28/1.2	29/1.3	5.1	PD/LBW	
		63.2	57.1			
10	MCD	23/0.9	25/1.0	3.2	NN	
		92.1	80.2			
11	2nd Preg	26/1.0	27/1.1	0.8	NN	
		79.7	70.6			
12	MN	24/1.0	26/1.0	8.2	NN	
		80.7	79.7			
13	2nd Preg	26/1.0	27/1.2	1.6	NN	
		79.7	63.3			
14	MPGN/CH	28/1.1	30/1.2	3.5	NN	
		70.2	62.5			
15	MN	22/0.9	23/1.1	3.1	PD/LBW	
		92.7	72.4			
16	2nd Preg	25/1.0	26/1.2	1.8	NN	
		80.2	64.0			
17	MCD	27/1.0	28/1.1	2.2	NN	
		79.2	70.2			
18	2nd Preg	31/1.0	33/1.1	3.6	NN	
		77.2	68.0			
19	LN III/CH	29/0.9	30/1.1	3.1	PD/LBW	PE
		88.7	69.3			
20	FSGS/CH	39/1.0	41/1.2	1.8	PD/LBW	
		73.5	58.3			
21	IgAN	33/0.8	35/0.9	3.7	NN	
		99.7	85.5			
22	HIVAN/CH	36/1.1	37/1.3	2.4	PD/LBW	PE
		66.8	54.3			
23	IgAN	31/0.9	33/0.9	0.7	NN	
		87.7	86.6			
24	LN IV	24/1.0	25/16	4.4	PM	PE
		80.7	45.6			
25	FSGS/CH	35/1.1	37/1.4	2.3	PD/LBW	PE
		67.2	49.7			
26	LN V	29/1.0	31/1.1	4.6	PD/LBW	
		78.2	68.9			
27	RN	32/1.0	34/1.1	0.9	NN	
		76.8	67.6			

Abbeviations – FSGS: focal segmental glomerulosclerosis, LN: lupus nephritis, IgAN: IgA nephropathy, MN: membranous nephropathy, MCD: minimal change disease, MPGN: membranoproliferative glomerulonephritis, HIVAN: HIV associated nephropathy, RN: reflux nephropathy. 2^nd^ Preg: second pregnancy, CH: chronic hypertension, Pu: proteinuria, HDc: chronic hemodialysis, NN: normal newborn, PM: perinatal mortality, PD: preterm delivery, LBW: low birth weight, PE: preeclampsia, PA: placental abruption. ESRD: end-stage renal disease.

**Table 2:** Clinical data and maternal fetal outcomes of women in Group II

N	Diseases pre-Pregnancy	Age/Scr CKDEPI-Pre	Age/Scr CKDEPI-Post	Greater Pu in Pregnancy	Fetal Outcome	Maternal Outcome
1	FSGS/CH	26 / 1.9	HDc	4.9	PD/LBW	PE/ESRD
		36.9				
2	LN V/CH	28/1.7	29/2.2	5.1	PM	
		41.6	30.4			
3	FSGS/CH	29/1.5	31/1.9	2.1	NN	
		48.1	35.8			
4	FSGS/CH	38/1.3	39/1.8	5.2	PD/LBW	
		54.0	36.3			
5	MPGN/CH	23/1.4	24/2.0	3.0	PD/LBW	PE
		54.2	35.1			
6	FSGS/CH	32/1.6	33/2.6	4.2	PD/LBW	PE
		43.7	24.2			
7	FSGS/CH	24/1.3	26/1.5	2.8	NN	
		58.9	49.0			
8	2nd Preg	29/1.6	30/2.1	3.0	PD/LBW	PE
		44.5	31.9			
9	MN	36/1.3	38/1.7	5.2	NN	
		54.7	39.1			
10	IgAN/CH	32/1.8	33/3.1	3.4	PM	PE
		37.9	19.6			
11	LN V/CH	30/1.3	31/1.9	4.0	PM	PA
		56.7	35,8			

Abbeviations – FSGS: focal segmental glomerulosclerosis, LN: lupus nephritis, IgAN: IgA nephropathy, MN: membranous nephropathy, MPGN: membranoproliferative glomerulonephritis. 2^nd^ Preg: second pregnancy, CH: chronic hypertension, Pu: proteinuria, HDc: chronic hemodialysis, NN: normal newborn, PM: perinatal mortality, PD: preterm delivery, LBW: low birth weight, PE: preeclampsia, PA: placental abruption. ESRD: end-stage renal disease.

**Table 3:** Clinical data and maternal fetal outcomes of pregnant women in Group III

N	Diseases pre-Pregnancy	Age/Scr CKDEPI-Pre	Age/Scr CKDEPI-Post	Greater Pu in Pregnancy	Fetal Outcome	Maternal Outcome
1	FSGS/CH	24/2.4	HDc	3.9	PD/LBW	PE/ESRD
		28.0				
2	LN IV/CH	19/3.1	HDc	4.2	PM	PA/ESRD
		21.4				
3	LN IV/CH	30/2.9	HDc	5.6	PM	PE/ESRD
		21.7				
4	AS/CH	25/2.8	HDc	4.0	PD/LBW	ESRD
		23.3				
5	FSGS/CH	18/3.2	HDc	5.1	PD/LBW	ESRD
		20.7				
6	HIVAN/CH	32/2.4	34/4.0	3.8	PD/LBW	PE
		26.8	14.4			

Abbeviations – FSGS: focal segmental glomerulosclerosis, LN: lupus nephritis, HIVAN: HIV associated nephropathy, AS: alport syndrome. CH: chronic hypertension, Pu: proteinuria, HDc: Chronic Hemodialysis. PM: perinatal mortality, PD: preterm delivery, LBW: low birth weight, PE: preeclampsia, PA: placental abruption, ESRD: end-stage renal disease.

In comparing the age and GD duration before conceiving among the groups, we only observed that Group II exhibited a significantly (p = 0.01) longer GD duration than Group I. During gestation, women with CH had their blood pressure controlled, except when superimposed preeclampsia occurred. Patients who experienced a GD flare or de novo GD diagnosed by renal biopsy or serological markers also presented NPu, except for one with LN II, and all of them required changes to their immunosuppression therapies.

During pregnancy, 8 patients progressed to ESRD and began hemodialysis: 2 (7.8%) in Group I (one with IgAN and the others with LN4) presented crescentic glomerulonephritis secondary to GD flare or de novo GD, 1 (9.0%) in group II with FSGS, and 5 (83.3%) in group III (2 with FSGS, 2 with LN IV and 1 with AS) likely due to GD progression.

**Figure 1:**
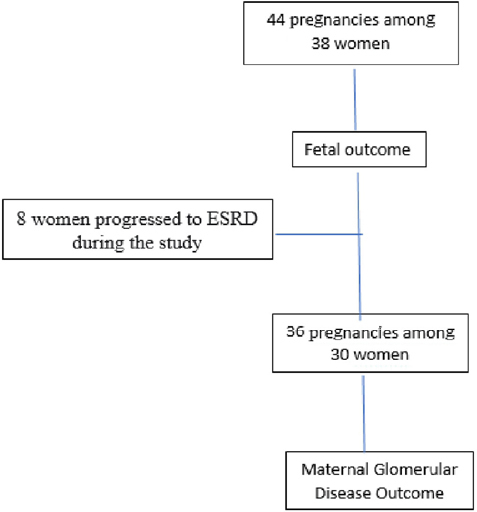
Flow diagram for patient inclusion.

We studied the fetal outcome in the 44 pregnancies among 38 women. However, for the GD progression study based on GFR, we only considered 36 pregnancies among 30 women. The analysis excluded 8 women who started hemodialysis during pregnancy (Figure 1).

### Fetal Outcome

The incidence of adverse fetal outcomes (intrauterine or neonatal death, preterm delivery, and low birth weight) among GD pregnant women was 63.6% (28/44) and the overall fetal mortality was 20.4% (9/44). In group I, adverse outcomes occurred in 51.8% (14/27) with intrauterine or neonatal death in 4/27 (14.8%) (Table 1); in group II adverse outcomes occurred in 72.7% (8/11) with intrauterine or neonatal death in 3/11 (27.2%) (Table 2), and in group III adverse outcomes occurred in 100% (6/6) with intrauterine or neonatal death in 2/6 (33.3%) (Table 3).

### Maternal Outcome

We found a higher incidence of adverse maternal outcomes in 52.2% (23/44) pregnancies (PE in 16, PA in 2, and ESRD in 8 women) and in 3 of these gestations, we observed both preeclampsia and progression to ESRD.

### Risk Factors for Maternal GD Progression In Pregnancy

The impact of pregnancy on the progression of GD was studied by comparing GFR before and one year after pregnancy. Out of the observed patients, 8 progressed to ESRD during pregnancy and started hemodialysis (2 in group I, 1 in group II, and 5 in group III). The remaining 36 pregnancies showed a significant (p < 0.0001) loss of GFR, with a difference of 13.4 ± 6.1 (range:1.0 to 35.1) mL/min/1.73m^2^ between GFR before (69.5 ± 17.4, range: 26.8 to 99.7) mL/min/1.73 m^2^ and one year after pregnancy (56.1 ± 19.1, range:14.4 to 86.6) mL/min/1.73 m^2^.

We also investigated the impact of risk factors. Our findings indicate that a low GFR level before conception and the presence of preeclampsia during gestation can lead to increased GFR loss (Table 4).

**Table 4:** Impact of risk factors in glomerular filtration rate loss

Risk Factor	GFR loss one year after pregnancy with	GFR loss one year after pregnancy without	p
Chronic hypertension	13.8 ± 4.2	12.6 ± 8.5	0.951
Nephrotic Proteinuria	13.6 ± 7.6	13.2 ± 4.8	0.899
Pre-eclampsia	17.0 ± 6.2	11.3 ± 5.1	0.001
Risk Factor	GFR loss one year after pregnancy in CKD Stage 1-2	GFR loss one year after pregnancy in CKD Stage 3a-3b	P
CKD KDIGO classification	12.5 ± 6.8	15.7 ± 3.9	0.013

Abbeviations – CKD: Chronic Kidney Disease, GFR: Glomerular Filtration rate.

GFR loss was significantly greater (p < 0.013) in Group II. Group I had a mean loss of 12.5 ± 6.8 (range: 1.0 to 35.1) mL/min/1.73m^2^ and Group II, of 15.7 ± 3.9 (range: 9.9 to 26.9) mL/min/1.73m^2^ (Table 4). In Group III: 5/ 6 (83.33%) progressed to ESRD.

Our study found a high incidence (13/36 (31.1%) of superimposed PE. Thirteen pregnancies with PE had an average GFR loss of 17.0 ± 6.2 (range: 11.0 to 35.9), while 23 pregnancies without PE had an average GFR loss of 11.3 ± 5.1 (range: 1.0 to 20.9) mL/min/1.73 m^2^. Superimposed PE significantly increased the GFR loss one year after pregnancy (p = 0.001).

We found that the presence of CH before conception did not impact GFR loss. We observed that in 22 pregnancies with CH, the GFR loss was 13.8 ± 4.2 (range: 6.1 to 20.9) mL/min/1.73 m^2^, while in 14 pregnancies without CH before conception, the GFR loss was 12.6 ± 8.5 (range: 1.0 to 35.1) mL/min/1.73 m^2^ (p = 0.951). However, the presence of CH significantly increased the risk of superimposed PE (p < 0.004, RR = 7.636 [1.111 < RR < 52.468]).

A high proteinuria peak was observed during gestation (mean 3.2 ± 1.5; range: 0.7 to 8.2 g/24 h), and 16/36 pregnancies (44.4%) showed NPu (mean 4.5 ± 1.1, range: 3.5 to 8.2 g/24 h), while the remaining 20/36 pregnancies (55.6%) exhibited Pu (mean 2.2 ± 0.8, range: 0.7 to 3.4 g/24 h).When comparing the GFR loss between pregnancies with NPu (GFR loss 13.6 ± 7.6, range: 1.0 to 35.19 mL/min/1.73 m^2^) and those without NPu (GFR loss 13.2 ± 4.8, range: 1.1 to 20.3 mL/min/1.73 m^2^), no significant difference was found (p = 0.899).

## DISCUSSION

Chronic kidney disease is a common health concern affecting many women of childbearing age. The medical community has actively researched the complex relationship between pregnancy and kidney disease, and this field is now known as “obstetric nephrology”. However, there is still limited understanding of how pregnancy may impact the progression of these diseases.

It’s important to detect glomerular disease (GD) early in pregnant women so that the pregnancy can be closely monitored. This helps to have a better understanding of how GD affects pregnancy, ultimately leading to improved maternal-fetal outcomes across different types and stages of these diseases. Therefore, we must include in all pregnancy guidelines simple and cost-effective laboratory tests such as dipstick urinalysis and serum creatinine testing before or at the beginning of pregnancy. These tests will help identify the presence of GD characterized by proteinuria or hematuria and determine kidney function^12^.

Women diagnosed with GD should have their urine samples tested for protein/creatinine ratio before and during pregnancy^13^. Monitoring proteinuria is essential for tracking GD progression during gestation. Improving our understanding of how different types and stages of GD affect maternal-fetal outcomes enables us to offer personalized preconception counseling to help women make informed decisions about pregnancy.

We conducted a study at a single center to investigate the effects of pregnancy on 38 women diagnosed with different types of primary and secondary GD through biopsy before conception. The most common type of GD was FSGS, found in 11 (25.5%), followed by LN in 10 (23.2%) and IgAN in 6 (13.9%) of the patients. The prevalence of GD was consistent with what has been reported in the literature, where lupus nephritis and FSGS are the most common GD types in Latin America^14^.

During pregnancy, eight patients progressed to ESRD and began hemodialysis. Two patients in CKD stage 1 or 2 presented rapidly progressive glomerulonephritis and required renal biopsy^15^ (one with IgAN at 16 and the other with LN IV at 18 weeks of gestation), and crescentic glomerulonephritis, which was secondary to GD flare or de novo GD, was diagnosed in both biopsies. In one patient with FSGS in CKD stage 3b, and five patients in CKD stage 4 (two with FSGS, two with LN IV, and one with AS), the progression to ESRD was likely due to GD progression associated with pregnancy-induced renal physiological alterations.

It has been observed that women with CKD who become pregnant at stages 3b, 4, or 5, are at a higher risk of CKD progression and may need to start a regular course of long-term dialysis during pregnancy to achieve uremic state control^16^. Therefore, it is recommended that women in these stages receive preconception counseling that includes a discussion of the possibility of needing dialysis during pregnancy. This can support better-informed shared decision-making^17^.

Pharmacologic therapeutic measures used to reduce glomerular hypertension and hyperfiltration must be stopped before conception, which result in increased proteinuria and increased risk of adverse pregnancy outcomes. Anti-proteinuric agents such as renin-angiotensin-aldosterone inhibitors and sodium-glucose transport-2 inhibitors (SGTL2) are teratogenic or fetotoxic, and a low-protein diet during pregnancy may cause significant alterations in fetal development and must therefore be discontinued^18^.

Glomerular hypertension and hyperfiltration on the remnant nephrons cause hypertrophy and ultimately proteinuria and glomerular sclerosis, and there is no validated drug approach to reduce protein leakage during pregnancy. Some studies have suggested that a plant-based diet with moderate protein restriction supplemented with alpha-keto analogs, amino acids, vitamins, and oligo elements may help stabilize proteinuria and uremia in GD pregnant women^19,20^. However, it’s always difficult to change dietary habits, especially in adults. Using vegetable protein supplements could be an option to ensure adequate protein intake during pregnancy.

### Impact of Risk Factors On Maternal GFR Loss

In our study, we also investigated the risk factors linked to an increased risk of maternal GD progression during pregnancy by GFR loss one year after giving birth (Table 4).

Out of 36 pregnancies, we found a significant loss of GFR (p < 0.0001) one year after childbirth. When comparing the groups, GFR loss was significantly higher (p < 0.013) in Group II than in Group I. In Group III, 5/6 (83.3%) patients progressed to ESRD during gestation. According to recent data from the UK, about 46% of pregnancies with stages 3 to 5 CKD experienced kidney function decline or started renal replacement therapy during pregnancy or within the first year after childbirth^16^. The risk of CKD progression is present at all stages of kidney disease, although absolute risk is low in early-stage CKD, and the risk increases as the disease advances^21^.

Our findings indicate that CH did not affect kidney disease progression in pregnant women with GD, and there was no significant impact on GFR loss (p = 0.951). Therefore, in our study, blood pressure was effectively controlled during pregnancy (≤ 140/90 mm Hg)^22^, except when superimposed PE occurred. However, the presence of CH increased the risk of developing superimposed PE by 7.6 times, which is similar to pregnant women without GD^23^.

It has been shown that the presence of Pu > 1 g/24 h and advanced stages of CKD are significant risk factors for adverse pregnancy outcomes^24,25^. Our study observed a high proteinuria peak during gestation (mean 3.2 ± 1.5; range: 0.7 to 8.2 g/24 h) in women with GD. However, the presence of NPu during pregnancy did not lead to a greater GFR loss (p = 0.89). GFR loss did not correlate with Pu severity during gestation in GD women with Pu > 1 g/24 hours, as observed in other studies^26^.

Preeclampsia is a pregnancy complication that affects 2 to 4% of pregnancies worldwide. It is a condition that progresses unpredictably, is characterized by glomerular endotheliosis associated with podocytopathy, and may be considered the most common GD globally^27^. Women with CKD are ten times more likely to develop PE than those without CKD, and the angiogenic markers, such as PlGF and sFlt-1 that can help diagnose superimposed pre-eclampsia, have been demonstrated to have limited diagnostic performance in women with CKD^28^.

Our study found a high incidence (31.1%) of superimposed PE among GD women. This was associated with a significant increase (p = 0.001) in GFR loss one year after pregnancy. Studies of long-term renal outcomes do not support the earlier notion that PE is a disease cured by delivery. PE increases the risk of future cardiovascular and renal diseases, and CKD risk is highest in proteinuric renal disease patients^29^.

### Fetal Outcomes

There is increasing evidence that even in the early stages of CKD, the risk of adverse fetal outcomes is higher than in the general population^30^. Our study showed that pregnancy in women with GD poses a high risk for adverse fetal outcomes, mainly in CKD advanced stages. We observed a higher overall rate of adverse fetal outcomes: 63.6% (28/44) in CKD stages 1 and 2, 72.7% (8/11) in CKD stages 3a and 3b, and 100% (6/6 gestations) in stages 4 and 5.

We observed a high overall fetal mortality (20.4%, 9/44 pregnancies), which was directly related to CKD stage. However, it has been demonstrated that the use of adequate intensive hemodialysis during pregnancy^11^, when clinically indicated to control the uremic state, may lead to reduced fetal mortality^31^.

Our study has some limitations. It was conducted at a single institution and included a small number of patients with various types of glomerular diseases. However, it’s important to note that all the patients had confirmed glomerular diseases before planning their pregnancies. They received careful monitoring during pregnancy from a specialized team and were followed up for at least one year after giving birth.

In conclusion, pregnancy is possible for women with GD, but it remains a challenging task for nephrologists and obstetricians worldwide. A successful pregnancy requires individualized preconception counseling, careful pregnancy planning, and a multidisciplinary approach involving nephrologists and high-risk obstetricians.

Our study indicates that GD increased maternal-fetal adverse outcomes and that the important changes in immunity and renal physiology secondary to gestation may act as a risk factor for GD progression. The loss of GFR secondary to pregnancy was directly associated with CKD stage before pregnancy and PE during pregnancy. Women mainly at CKD stage 4 or 5 should receive individualized and comprehensive preconception counseling, including a discussion regarding the potential GD progression, the need for dialysis during gestation, and maternal-fetal outcomes.

Although planned pregnancy has improved maternal-fetal outcomes for these women in recent years, the effects of the interaction between GD and pregnancy extend beyond delivery. They can impact future cardiovascular and renal health of both the mothers and their potentially preterm underweight babies.

## Data Availability

The complete dataset that underlies the results of this study has been published in the article itself.
