# Supplementary material for: The effects of pregnancy on the progression of maternal glomerular disease
Source: J Bras Nefrol. 2025 Aug 1;47(4):e20240205. doi: 10.1590/2175-8239-JBN-2024-0205en (PMC12321267; doi:10.1590/2175-8239-JBN-2024-0205en)
Supplement: Translation

# Translation: Os efeitos da gravidez na progressão da doença glomerular materna

**DOI:** 10.1590/2175-8239-JBN-2024-0205pt

**Authors:** Luiz Paulo José Marques, Lívia Menezes Salla, Lilimar da Silveira Rioja, Regina Rocco, Eugênio Pacelle Queiroz Madeira, Lygia Maria Soares Fernandes Vieira

**Affiliations:** 1 Universidade Federal do Estado do Rio de Janeiro, Hospital Universitário Gaffrée e Guinle, Departamento de Medicina, Rio de Janeiro, RJ, Brasil.

**Keywords:** Insuficiência Renal Crônica, Doenças Glomerulares, Gravidez, Hiper­tensão Crônica, Proteinúria, Pré-Eclâmpsia

## Abstract

**Introdução:**

Embora a maioria das mulheres com doenças glomerulares (DG) subjacentes esteja em idade reprodutiva, existem poucas informações sobre como a gravidez afeta essas condições e os desfechos maternos.

**Métodos:**

Estudo de coorte retrospectivo, de centro único, envolvendo 44 gestações planejadas em 38 pacientes com DG comprovada por biópsia. Dividiu-se as pacientes em três grupos conforme classificação DRC-KDIGO pré-concepção: I) Estágios 1–2: 27 gestações, II) Estágios 3a–3b: 11 gestações, III) Estágios 4–5: 6 gestações. Dados clínicos incluíram idade, hipertensão crônica (HC), creatinina sérica, pré-eclâmpsia (PE), proteinúria. Consideramos HC, estágio da DRC antes da gravidez e PE e proteinúria nefrótica (PNu) durante a gestação como fatores de risco para progressão da DG materna.

**Resultados:**

8 mulheres evoluíram para DRET e iniciaram hemodiálise durante a gravidez: Grupo I – 2 (7,8%); Grupo II – 1 (9,0%); Grupo III – 5 (83,3%). Nas 36 gestações restantes, observamos perda significativa de TFG (p < 0,0001) um ano após a gravidez, com maior perda no grupo II do que no I (p < 0,013). Taxas reduzidas de TFG antes da gravidez e PE durante a gravidez (p = 0,001) impactaram diretamente na perda de TFG. Também observamos alta incidência (63,6%) de desfechos fetais adversos.

**Conclusão:**

Embora a gestação seja possível para mulheres com DG, seu impacto na DG materna continua após o parto. Ter DG aumenta os riscos de desfechos gestacionais adversos. A progressão da DG relaciona-se diretamente com estágio da DRC antes da gravidez e PE durante a gravidez. Mulheres com DRC estágios 4–5 apresentam alto risco de progredir para DRET na gestação.

## Introdução

A doença renal crônica (DRC) é um fator de risco para desfechos gestacionais adversos, mesmo nos estágios iniciais da doença. A DRC deve ser incluída entre os fatores de risco significativos, como diabetes e hipertensão^1^. Estudos têm demonstrado que 3 a 6% das mulheres em idade reprodutiva apresentam DRC, e até 3,3% das gestantes têm evidências laboratoriais de DRC^2,3^. O efeito da gravidez na doença renal e o impacto da doença renal na gravidez podem ser prejudiciais.

Embora a maioria das mulheres com doenças glomerulares (DG) subjacentes esteja em idade reprodutiva, essas doenças podem passar desperce­bidas durante a gravidez e só ser diagnosticadas em fases posteriores da vida. Um estudo australiano diagnosticou 0,3% das mulheres grávidas com doenças renais; doenças imunológicas ou doenças glomerulares só foram identificadas em 0,01%^4^.

A gestação provoca alterações importantes na imunidade e na fisiologia renal, o que pode acelerar a progressão da DG. Essas alterações incluem a diminuição da resistência vascular renal, associada a elevações no débito cardíaco e no fluxo sanguíneo renal, com um aumento simultâneo de 38 a 56% na taxa de filtração glomerular (TFG)^5^. Isso resulta em um aumento fisiológico na excreção de proteínas em decorrência do aumento da TFG e da permeabilidade da membrana basal glomerular^6^. Outra adaptação importante é a modulação das imunidades inata e adaptativa para estabelecer a tolerância materna ao feto semi-alogênico, que expressa antígenos maternos e paternos^7^.

Nas últimas décadas, tem havido uma atenção crescente às complexas interações entre a DRC e a gravidez, bem como aos diversos aspectos da gestação em mulheres com doenças renais. No entanto, pouco se sabe sobre doenças glomerulares específicas além do lúpus e da nefropatia por IgA, que ocorrem com mais frequência^8,9^.

As DG comumente afetam mulheres em idade reprodutiva e podem representar muitos desafios durante a gravidez, incluindo doenças imunológicas, hipertensão, proteinúria e lesão do tecido renal. No entanto, há informações limitadas sobre os desfechos dessas condições, particularmente no que se refere à progressão da doença renal materna. É essencial ampliar esse conhecimento para aprimorar o aconselhamento pré-concepcional personalizado e abrangente, melhorar os desfechos materno-fetais e personalizar a prática clínica.

Portanto, nosso objetivo foi avaliar o impacto da gravidez na progressão das DG. Estudamos os efeitos da gestação na função renal, os fatores de risco para desfechos maternos adversos e os desfechos materno-fetais dessas pacientes.

## Métodos

### Sujeitos do Estudo

Este estudo foi realizado com gestantes que apresentaram DG confirmada por biópsia antes da concepção e receberam atendimento obstétrico pré-natal e nefrológico no Hospital Universitário Gaffrée e Guinle de janeiro de 2009 a dezembro de 2019. Todas as participantes forneceram voluntariamente um termo de consentimento informado por escrito. Esse estudo clínico foi realizado em conformidade com a Declaração de Helsinque e aprovado pelo comitê local de ética em pesquisa em seres humanos, sob o número de aprovação: 3.046.179.

Foram excluídas do estudo as mulheres com nefropatia diabética devido aos desfechos materno-fetais peculiares relacionados ao diabetes^10^ e aquelas que apresentaram crises de DG ou DG *de novo* no primeiro ano após o parto.

Todas as pacientes tiveram uma gravidez planejada e foram submetidas a um monitoramento rigoroso por uma equipe multiprofissional especializada. Um protocolo predefinido de avaliação laboratorial e clínica foi seguido em visitas programadas durante toda a gestação^11^.

### Desenho do Estudo

Este estudo de coorte retrospectivo, descritivo e de centro único foi realizado com pacientes grávidas com DG primária ou secundária, que tiveram pelo menos 12 meses de acompanhamento após o parto em nosso hospital. A TFG foi medida por meio da equação CKD-EPI antes da concepção e 12 meses após o parto. As pacientes foram divididas em três grupos, de acordo com a classificação KDIGO para DRC antes da gravidez: Grupo I (estágios 1-2): TFG ≥ 60 mL/min/1,73m^2^, Grupo II (estágios 3a-3b): TFG ≤ 59 e ≥ 30 mL/min/1,73m^2^ e Grupo III (estágios 4-5): TFG ≤ 29 mL/min/1,73m^2^.

### Coleta de Dados Clínicos

Foram coletados os seguintes dados clínicos: idade, resultados de biópsia renal, tempo de diagnóstico de DG, pressão arterial, uso de terapia anti-hipertensiva e imunossupressora e desfechos materno-fetais.

Os parâmetros laboratoriais registrados incluíram: ureia, creatinina sérica (CrS) antes da concepção e doze meses após o parto, maior proteinúria (Pu) durante a gravidez (os níveis de proteína urinária foram mensurados na décima segunda, vigésima, trigésima e trigésima sexta semanas de gestação ou quando necessário, por meio de coleta de urina de 24 horas), complemento sérico (C3 e C4), anti-DNA de fita dupla e anti-PLA2R. A equação CKD-EPI foi calculada utilizando o site https://qxmd.com, versão 2021. A classificação ISN/RPS 2018 para nefrite lúpica foi aplicada.

### Desfechos Materno-Fetais

Os desfechos fetais adversos incluíram mortalidade perinatal (MP, óbito de um feto viável após 22 semanas de gestação ou dentro de 4 semanas após o parto, incluindo natimorto), parto prematuro (PP, parto antes de 37 semanas de gestação) e baixo peso ao nascer (BPN, peso do bebê inferior a 2500 g ao nascimento). Recém-nascido normal (RN) é o termo que se refere a um bebê que nasceu a termo, estável e saudável.

Os desfechos maternos adversos incluíram pré-eclâmpsia (PE, definida como hipertensão arterial (HA) acompanhada de piora da Pu após 20 semanas de gestação e, para mulheres com HA preexistente, a PE foi definida como piora da condição aumento da pressão arterial sistólica em 30 mm Hg e da pressão arterial diastólica em 15 mm Hg associada à piora da Pu após 20 semanas de gestação), descolamento prematuro da placenta (DPP, ocorre quando há separação parcial ou total da placenta da parede interna do útero antes do parto) e progressão para doença renal em estágio terminal (DRET, necessidade de um curso regular de diálise de longo prazo).

### Fatores de Risco Para A Progressão da Doença Glomerular Materna na Gravidez

Hipertensão crônica (HC) e estágios KDIGO mais avançados de DRC antes da concepção, além de PE e proteinúria nefrótica (PNu) durante a gravidez, foram considerados fatores de risco para a progressão da doença glomerular materna.

A HC foi definida como pressão arterial sistólica >140 e pressão arterial diastólica >90, ou pacientes em terapia anti-hipertensiva antes da concepção. A PNu foi definida como Pu ≥ 3,5 g/24 h. Estágio DRC-KDIGO: Classificação do estágio KDIGO da Doença Renal Crônica, de 1 a 5.

### Análise Estatística

A análise estatística foi realizada no GraphPad InStat-3 (GraphPad Software, San Diego, CA). Os dados categóricos e numéricos foram expressos como médias ± desvios padrão; um teste T ou análise de variância (ANOVA) foi utilizado para comparação dos dados. Os dados de desfecho foram comparados utilizando o teste de pares combinados de Wilcoxon, e as correlações bivariadas dos fatores de risco entre os grupos foram analisadas com o teste exato de Fisher. O risco relativo (RR) e os intervalos de confiança de 95% foram calculados por meio de métodos padrão. Um valor de P < 0,05 foi considerado estatisticamente significativo.

## RESULTADOS

Este estudo incluiu 38 mulheres com idade média de 28,1 ± 4,7 (intervalo: 18 a 39) anos, que tiveram DG de longa duração diagnosticada de 18 a 172 (média de 62,3 ± 31,6) meses, por meio de biópsia renal, antes da concepção: 11 casos de glomeruloesclerose segmentar focal (GESF) com 12 gestações, 10 casos de nefrite lúpica (NL) com 11 gestações, 6 casos de nefropatia por IgA (NIgA), 3 casos de nefropatia membranosa (NM) com 5 gestações, 2 casos de doença de lesão mínima (DLM) com 4 gestações, 2 casos de glomerulonefrite membranoproliferativa (GNMP), 2 casos de nefropatia associada ao HIV (NAHIV), 1 caso de síndrome de Alport (SA) e 1 caso de nefropatia de refluxo (NR) associada à nefropatia esclerosante.

As pacientes foram acompanhadas por pelo menos 12 meses após o parto. Das 38 pacientes, 27 (71%) eram da nossa Unidade de Nefrologia, enquanto 11 (29%) foram encaminhadas de outros hospitais durante o primeiro ou segundo trimestre de gravidez. Todas as gestações foram planejadas e os medica­mentos teratogênicos utilizados para hipertensão, HIV e imunossupressão foram substituídos por alternativas terapêuticas mais seguras antes da concepção e durante as crises de DG ou DG *de novo*.

A presença de PNu (3,5 ± 1,3, variando de 0,7 a 8,2 g/24) foi observada em 16 gestações, 8 das quais apresentaram crises de DG ou sinais de DG *de novo*. Dentre essas, 2 gestantes com NM apresentaram níveis elevados de anti-PLA2R, 4 gestantes com NL apresentaram aumento do anti-DNA de fita dupla associado à redução do complemento sérico e os 2 casos restantes foram confirmados por meio de biópsia renal durante a gravidez.

No entanto, as medições de proteinúria só foram obtidas antes da concepção em 16 das 44 gestações.

### Dados Clínicos das Gestantes

As mulheres foram divididas em três grupos de acordo com a classificação KDIGO para DRC. No Grupo I (Estágios 1–2), constavam 22 (57,9%) pacientes com 27 (61,3%) gestações, com idade de 28,2 ± 4,3 (22 a 39) anos e tempo de diagnóstico de DG de 61,1 ± 32,6 (intervalo: 22 a 172) meses (Tabela 1). No Grupo II (Estágios 3a–3b), havia 10 (26,3%) pacientes com 11 (25%) gestações com idade de 29,7 ± 4,6 (23 a 38) anos e tempo de diagnóstico de DG de 77,3 ± 27,6 (intervalo: 47 a 122) meses (Tabela 2). No Grupo III (Estágios 4–5), havia 6 (15,8%) pacientes com 6 (13,7%) gestações com idade de 24,6 ± 5,6 (18 a 32) anos e tempo de diagnóstico de DG de 47,0 ± 27,2 (intervalo: 18 a 94) meses (Tabela 3).

**Tabela 1:** Dados clínicos e desfechos materno-fetais das gestantes no Grupo I

N	Doenças pré -gestacionais	Idade/CrS CKD-EPI-Pré	Idade/CrS CKD-EPI-Pós	Maior Pu na Gravidez	Desfecho Fetal	Desfecho Maternal
1	NL IV	29/1,1	HDc	6,1	MP	DRET
		69,8				
2	NIgA	22/0,9	HDc	4,2	MP	DRET
		92,7				
3	NL IV/HC	26/1,2	27/1,4	3,6	MP	PE
		64,0	52,9			
4	GESF/HC	31/1,0	33/1,2	3,9	PP/BPN	PE
		77,2	61,3			
5	NIgA/HC	30/0,9	32/1,0	4,6	PP/BPN	PE
		88,2	76,8			
6	NIgA/HC	22/1,0	23/1,2	2,9	PP/BPN	PE
		81,7	65,2			
7	NL II/HC	26/1,0	28/1,1	2,4	RN	
		79,7	70,2			
8	2^a^gravidez	29/1,0	30/1,1	1,0	RN	
		78,2	69,3			
9	GESF/HC	28/1,2	29/1,3	5,1	PP/BPN	
		63,2	57,1			
10	DLM	23/0,9	25/1,0	3,2	RN	
		92,1	80,2			
11	2ª gravidez	26/1,0	27/1,1	0,8	RN	
		79,7	70,6			
12	NM	24/1,0	26/1,0	8,2	RN	
		80,7	79,7			
13	2ª gravidez	26/1,0	27/1,2	1,6	RN	
		79,7	63,3			
14	GNMP/HC	28/1,1	30/1,2	3,5	RN	
		70,2	62,5			
15	NM	22/0,9	23/1,1	3,1	PP/BPN	
		92,7	72,4			
16	2ª gravidez	25/1,0	26/1,2	1,8	RN	
		80,2	64,0			
17	DLM	27/1,0	28/1,1	2,2	RN	
		79,2	70,2			
18	2ª gravidez	31/1,0	33/1,1	3,6	RN	
		77,2	68,0			
19	NL III/HC	29/0,9	30/1,1	3,1	PP/BPN	PE
		88,7	69,3			
20	GESF/HC	39/1,0	41/1,2	1,8	PP/BPN	
		73,5	58,3			
21	NIgA	33/0,8	35/0,9	3,7	RN	
		99,7	85,5			
22	NAHIV/HC	36/1,1	37/1,3	2,4	PP/BPN	PE
		66,8	54,3			
23	NIgA	31/0,9	33/0,9	0,7	RN	
		87,7	86,6			
24	NL IV	24/1,0	25/16	4,4	MP	PE
		80,7	45,6			
25	GESF/HC	35/1,1	37/1,4	2,3	PP/BPN	PE
		67,2	49,7			
26	NL V	29/1,0	31/1,1	4,6	PP/BPN	
		78,2	68,9			
27	NR	32/1,0	34/1,1	0,9	RN	
		76,8	67,6			

Abreviação – GESF: glomeruloesclerose segmentar focal, NL: nefrite lúpica, NIgA: Nefropatia por IgA, NM: nefropatia membranosa, DLM: doença de lesão mínima, GNMP: glomerulonefrite membranoproliferativa, NAHIV: Nefropatia associada ao HIV, NR: nefropatia de refluxo. HC: hipertensão crônica, Pu: proteinúria, HDc: hemodiálise crônica, RN: recém-nascido normal, MP: mortalidade perinatal, PP: parto prematuro, BPN: baixo peso ao nascer, PE: pré-eclâmpsia, DPP: descolamento prematuro da placenta. DRET: doença renal em estágio terminal.

**Tabela 2:** Dados clínicos e desfechos materno-fetais das gestantes no Grupo II

N	Doenças pré- gestacionais	Idade/CrS CKD-EPI-Pré	Idade/CrS CKD-EPI-Pós	Maior Pu na Gravidez	Desfecho Fetal	Desfecho Maternal
1	GESF/HC	26/1,9	HDc	4,9	PP/BPN	PE/DRET
		36,9				
2	NL V/HC	28/1,7	29/2,2	5,1	MP	
		41,6	30,4			
3	GESF/HC	29/1,5	31/1,9	2,1	RN	
		48,1	35,8			
4	GESF/HC	38/1,3	39/1,8	5,2	PP/BPN	
		54,0	36,3			
5	GNMP/HC	23/1,4	24/2,0	3,0	PP/BPN	PE
		54,2	35,1			
6	GESF/HC	32/1,6	33/2,6	4,2	PP/BPN	PE
		43,7	24,2			
7	GESF/HC	24/1,3	26/1,5	2,8	RN	
		58,9	49,0			
8	2ª gravidez	29/1,6	30/2,1	3,0	PP/BPN	PE
		44,5	31,9			
9	NM	36/1,3	38/1,7	5,2	RN	
		54,7	39,1			
10	NIgA/HC	32/1,8	33/3,1	3,4	MP	PE
		37,9	19,6			
11	NL V/HC	30/1,3	31/1,9	4,0	MP	DPP
		56,7	35,8			

Abreviação – GESF: glomeruloesclerose segmentar focal, NL: nefrite lúpica, NIgA: Nefropatia por IgA, NM: nefropatia membranosa, GNMP: glomerulonefrite membranoproliferativa. HC: hipertensão crônica, Pu: proteinúria, HDc: hemodiálise crônica, RN: recém-nascido normal, MP: mortalidade perinatal, PP: parto prematuro, BPN: baixo peso ao nascer, PE: pré-eclâmpsia, DPP: descolamento prematuro da placenta. DRET: doença renal em estágio terminal.

**Tabela 3:** Dados clínicos e desfechos materno-fetais das gestantes no Grupo III

N	Doenças pré- gestacionais	Idade/CrS CKD-EPI-Pré	Idade/CrS CKD-EPI-Pós	Maior Pu na Gravidez	Desfecho Fetal	Desfecho Maternal
1	GESF/HC	24/2,4	HDc	3,9	PP/BPN	PE/DRET
		28,0				
2	NL IV/HC	19/3,1	HDc	4,2	MP	DPP/DRET
		21,4				
3	NL IV/HC	30/2,9	HDc	5,6	MP	PE/DRET
		21,7				
4	SA/HC	25/2,8	HDc	4,0	PP/BPN	DRET
		23,3				
5	GESF/HC	18/3,2	HDc	5,1	PP/BPN	DRET
		20,7				
6	NAHIV/HC	32/2,4	34/4,0	3,8	PP/BPN	PE
		26,8	14,4			

Abreviação – GESF: glomeruloesclerose segmentar focal, NL: nefrite lúpica, NAHIV: Nefropatia por IgA, SA: Síndrome de Alport. HC: hipertensão crônica, Pu: proteinúria, HDc: hemodiálise crônica. MP: mortalidade perinatal, PP: parto prematuro, BPN: baixo peso ao nascer, PE: pré-eclâmpsia, DPP: descolamento prematuro da placenta, DRET: doença renal em estágio terminal.

Ao comparar a idade e a duração da DG antes da concepção entre os grupos, observamos apenas que o Grupo II apresentou uma duração significativamente maior (p = 0,01) de DG em relação ao Grupo I. Durante a gestação, as mulheres com HC tiveram sua pressão arterial controlada, exceto quando ocorreu pré-eclâmpsia sobreposta. As pacientes que apresentaram crises de DG ou DG *de novo* diagnosticada por biópsia renal ou marcadores sorológicos também apresentaram PNu exceto uma paciente com NL II – e todas elas necessitaram de mudanças em suas terapias de imunossupressão.

Durante a gravidez, 8 pacientes evoluíram para DRET e iniciaram hemodiálise: 2 (7,8%) no Grupo I (uma com NIgA e as demais com NL IV) apresentaram glomerulonefrite crescêntica secundária à exacerbação da DG ou DG *de novo*, 1 (9,0%) no Grupo II com GESF e 5 (83,3%) no Grupo III (2 com GESF, 2 com NL IV e 1 com SA) provavelmente devido à progressão da DG.

Estudamos os desfechos fetais nas 44 gestações de 38 mulheres. Entretanto, para o estudo da progressão da DG com base na TFG, consideramos apenas 36 gestações entre 30 mulheres. A análise excluiu 8 mulheres que iniciaram hemodiálise durante a gravidez (Figura 1).

**Figura 1:** Diagrama de fluxo para inclusão de pacientes.

### Desfechos Fetais

A incidência de desfechos fetais adversos (óbito intrauterino ou neonatal, parto prematuro e baixo peso ao nascer) entre as gestantes com DG foi de 63,6% (28/44) e a mortalidade fetal geral foi de 20,4% (9/44). No Grupo I, os desfechos adversos ocorreram em 51,8% (14/27) das gestações, com óbito intrauterino ou neonatal em 4/27 (14,8%) (Tabela 1); no Grupo II, os desfechos adversos ocorreram em 72,7% (8/11), com óbito intrauterino ou neonatal em 3/11 (27,2%) (Tabela 2) e no Grupo III, os desfechos adversos ocorreram em 100% (6/6), com óbito intrauterino ou neonatal em 2/6 (33,3%) (Tabela 3).

### Desfechos Maternos

Constatamos uma maior incidência de desfechos maternos adversos em 52,2% (23/44) das gestações (PE em 16, DPP em 2 e DRET em 8 mulheres). Em 3 dessas gestações, observamos tanto a pré-eclâmpsia quanto a progressão para DRET.

### Fatores de Risco Para A Progressão da DG Materna na Gravidez

O impacto da gestação na progressão da DG foi avaliado por meio da comparação da TFG antes e um ano após a gravidez. Das pacientes observadas, 8 progrediram para DRET durante a gravidez e iniciaram hemodiálise (2 no grupo I, 1 no grupo II e 5 no grupo III). As 36 gestações restantes apresentaram uma perda significativa (p < 0,0001) da TFG, com uma diferença de 13,4 ± 6,1 (intervalo: 1,0 a 35,1) mL/min/1,73m^2^ entre os valores de TFG antes (69,5 ± 17,4; intervalo: 26,8 a 99,7) mL/min/1,73 m^2^ e um ano após a gravidez (56,1 ± 19,1; intervalo: 14,4 a 86,6) mL/min/1,73 m^2^.

Também investigamos o impacto dos fatores de risco. Nossos achados indicam que níveis reduzidos de TFG antes da concepção e a presença de pré-eclâmpsia durante a gestação podem resultar em maior perda de TFG (Tabela 4).

**Tabela 4:** Impacto dos fatores de risco na perda da taxa de filtração glomerular

Fator de Risco	Perda de TFG um ano após a gravidez com	Perda de TFG um ano após a gravidez sem	p
Hipertensão crônica	13,8 ± 4,2	12,6 ± 8,5	0,951
Proteinúria nefrótica	13,6 ± 7,6	13,2 ± 4,8	0,899
Pré-eclâmpsia	17,0 ± 6,2	11,3 ± 5,1	0,001
Fator de Risco	Perda de TFG um ano após a gestação nos estágios 1-2 da DRC	Perda de TFG um ano após a gestação nos estágios 3a-3b da DRC	P
Classificação DRC-KDIGO	12,5 ± 6,8	15,7 ± 3,9	0,013

Abreviação – DRC: Doença Renal Crônica, TFG: Taxa de Filtração Glomerular.

A perda da TFG foi significativamente maior (p < 0,013) no Grupo II. O Grupo I apresentou uma perda média de 12,5 ± 6,8 (intervalo: 1,0 a 35,1) mL/min/1,73m^2^, enquanto o Grupo II apresentou uma perda média de 15,7 ± 3,9 (intervalo: 9,9 a 26,9) mL/min/1,73m^2^ (Tabela 4). No Grupo III: 5/6 (83,33%) evoluíram para DRET.

Nosso estudo encontrou uma alta incidência (13/36 [31,1%]) de PE sobreposta. Treze gestações com PE apresentaram uma perda média de TFG de 17,0 ± 6,2 (intervalo: 11,0 a 35,9), enquanto 23 gestações sem PE apresentaram uma perda média de TFG de 11,3 ± 5,1 (intervalo: 1,0 a 20,9) mL/min/1,73 m^2^. A PE sobreposta aumentou significativamente a perda de TFG um ano após a gravidez (p = 0,001).

Verificamos que a presença de HC antes da concepção não teve impacto sobre a perda de TFG. Observamos que em 22 gestações com HC, a perda de TFG foi de 13,8 ± 4,2 (intervalo: 6,1 a 20,9) mL/min/1,73 m^2^, enquanto em 14 gestações sem HC antes da concepção, a perda de TFG foi de 12,6 ± 8,5 (intervalo: 1,0 a 35,1) mL/min/1,73 m^2^ (p = 0,951). No entanto, a presença de HC aumentou significativamente o risco de PE sobreposta (p < 0,004; RR = 7,636 [1,111 < RR < 52,468]).

Um pico elevado de proteinúria foi observado durante a gestação (média de 3,2 ± 1,5; intervalo: 0,7 a 8,2 g/24 h), e 16/36 gestações (44,4%) apresentaram PNu (média 4,5 ± 1,1, intervalo: 3,5 a 8,2 g/24 h), enquanto as 20/36 gestações restantes (55,6%) apresentaram Pu (média 2,2 ± 0,8, intervalo: 0,7 a 3,4 g/24 h). Ao comparar a perda de TFG entre gestações com PNu (perda de TFG 13,6 ± 7,6, intervalo: 1,0 a 35,19 mL/min/1,73 m^2^) e aquelas sem PNu (perda de TFG 13,2 ± 4,8, intervalo: 1,1 a 20,3 mL/min/1,73 m^2^), não foi encontrada diferença significativa (p = 0,899).

## Discussão

A doença renal crônica é um problema de saúde comum que afeta muitas mulheres em idade fértil. A comunidade médica tem pesquisado ativamente a complexa relação entre gravidez e doença renal, área atualmente conhecida como “nefrologia obstétrica”. No entanto, ainda há um entendimento limitado sobre como a gestação pode afetar a progressão dessas doenças.

É importante detectar a doença glomerular (DG) precocemente em gestantes para que a gravidez possa ser monitorada rigorosamente. Com isso, é possível entender melhor como a DG afeta a gravidez, o que, em última análise, resulta em melhores desfechos materno-fetais nos diferentes tipos e estágios dessas doenças. Portanto, devemos incluir em todas as diretrizes de acompanhamento gestacional a realização de exames laboratoriais simples e de baixo custo, como a análise de urina com tira reagente e o teste de creatinina sérica, antes ou no início da gravidez. Esses exames ajudarão a identificar a presença de DG caracterizada por proteinúria ou hematúria e a determinar a função renal^12^.

Mulheres diagnosticadas com DG devem ter suas amostras de urina analisadas quanto à relação proteína/creatinina antes e durante a gestação^13^. O monitoramento da proteinúria é essencial para acompanhar a progressão da DG durante a gestação. Melhorar nossa compreensão de como os diferentes tipos e estágios da DG afetam os desfechos materno-fetais nos permite oferecer aconselhamento pré-concepcional personalizado, ajudando as mulheres a tomar decisões informadas sobre a gravidez.

Realizamos um estudo de centro único com o objetivo de investigar os efeitos da gravidez em 38 mulheres diagnosticadas com diferentes tipos de DG primária e secundária por meio de biópsia antes da concepção. O tipo mais comum de DG foi a GESF, encontrada em 11 (25,5%) pacientes, seguida por NL em 10 (23,2%) e NIgA em 6 (13,9%) pacientes. A prevalência de DG foi consistente com o que tem sido relatado na literatura, onde a nefrite lúpica e a GESF são descritas como os tipos mais comuns de DG na América Latina^14^.

Durante a gravidez, oito pacientes evoluíram para DRET e iniciaram hemodiálise. Duas pacientes em estágio 1 ou 2 da DRC apresentaram glomerulonefrite rapidamente progressiva e precisaram de biópsia renal^15^ (uma com NIgA na 16ª semana e outra com NL IV na 18ª semana de gestação). A glomerulonefrite crescêntica, secundária à exacerbação da DG ou à DG *de novo*, foi diagnosticada em ambas as biópsias. Em uma paciente com GESF no estágio 3b da DRC e em cinco pacientes no estágio 4 da DRC (duas com GESF, duas com NL IV e uma com SA), a progressão para DRET foi provavelmente atribuída à progressão da DG associada a alterações fisiológicas renais induzidas pela gestação.

Foi observado que mulheres com DRC que engravidam nos estágios 3b, 4 ou 5 apresentam um risco maior de progressão da DRC e podem precisar iniciar um tratamento regular de diálise de longo prazo durante a gestação, a fim de controlar o estado urêmico^16^. Portanto, recomenda-se que as mulheres nesses estágios recebam aconselhamento pré-concepcional que inclua uma discussão sobre a possibilidade de necessidade de diálise durante a gravidez. Isso pode apoiar uma tomada de decisão compartilhada e mais bem informada^17^.

As medidas terapêuticas farmacológicas utilizadas para reduzir a hipertensão glomerular e a hiperfiltração devem ser interrompidas antes da concepção, o que resulta em aumento da proteinúria e maior risco de desfechos gestacionais adversos. Agentes antiproteinúricos, como inibidores do sistema renina-angiotensina-aldosterona e inibidores do cotransportador de sódio-glicose tipo 2 (SGTL2), são teratogênicos ou fetotóxicos e devem ter seu uso interrompido. Além disso, uma dieta com baixo teor proteico durante a gravidez pode causar alterações significativas no desenvolvimento fetal devendo, portanto, ser descontinuada^18^.

A hipertensão glomerular e a hiperfiltração nos néfrons remanescentes causam hipertrofia e, por fim, proteinúria e esclerose glomerular, e não há nenhuma abordagem farmacológica validada para reduzir o vazamento de proteínas na urina durante a gravidez. Alguns estudos sugeriram que uma dieta baseada em vegetais com restrição moderada de proteínas, suplementada com alfa-cetoanálogos, aminoácidos, vitaminas e oligoelementos, pode ajudar a estabilizar a proteinúria e a uremia em gestantes com DG^19,20^. No entanto, mudar os hábitos alimentares é sempre um desafio, principalmente em adultos. O uso de suplementos de proteína vegetal pode ser uma opção para garantir a ingestão proteica adequada durante a gravidez.

### Impacto dos Fatores de Risco na Perda de TFG Materna

Em nosso estudo, também investigamos os fatores de risco associados a um risco maior de progressão da DG materna durante a gravidez por perda de TFG um ano após o parto (Tabela 4).

Em 36 gestações, encontramos uma perda significativa de TFG (p < 0,0001) um ano após o parto. Ao comparar os grupos, a perda de TFG foi significativamente maior (p < 0,013) no Grupo II em relação ao Grupo I. No Grupo III, 5/6 (83,3%) pacientes evoluíram para DRET durante a gestação. De acordo com dados recentes do Reino Unido, cerca de 46% das gestantes com DRC nos estágios 3 a 5 apresentaram declínio da função renal ou iniciaram terapia renal substitutiva durante a gravidez ou no primeiro ano após o parto^16^. O risco de progressão da DRC está presente em todos os estágios da doença renal, embora o risco absoluto seja baixo nos estágios iniciais. Esse risco aumenta à medida que a doença avança^21^.

Nossos achados indicam que a HC não afetou a progressão da doença renal em gestantes com DG, e não houve impacto significativo na perda de TFG (p = 0,951). Portanto, em nosso estudo, a pressão arterial foi controlada de forma eficaz durante a gestação (≤ 140/90 mm Hg)^22^, exceto quando ocorreu PE sobreposta. No entanto, a presença de HC aumentou o risco de desenvolver PE sobreposta em 7,6 vezes, valor semelhante ao de gestantes sem DG^23^.

Foi demonstrado que a presença de Pu >1 g/24 h e estágios avançados de DRC constituem fatores de risco significativos para desfechos gestacionais adversos^24,25^. Nosso estudo observou um alto pico de proteinúria durante a gestação (média de 3,2 ± 1,5; intervalo: 0,7 a 8,2 g/24 h) em mulheres com DG. No entanto, a presença de PNu durante a gravidez não levou a uma maior perda de TFG (p = 0,89). A perda de TFG não se correlacionou com a gravidade da Pu durante a gestação em mulheres com DG com Pu > 1 g/24 horas, como observado em outros estudos^26^.

A pré-eclâmpsia é uma complicação gestacional que afeta de 2 a 4% das gestações mundialmente. É uma condição que progride de forma imprevisível, caracterizada por endoteliose glomerular associada à podocitopatia, e pode ser considerada a DG mais comum em todo o mundo^27^. Mulheres com DRC apresentam uma probabilidade aproximadamente dez vezes maior de desenvolver PE em comparação àquelas sem a doença. Além disso, marcadores angiogênicos – como PlGF e sFlt-1 – que podem contribuir para o diagnóstico de pré-eclâmpsia sobreposta, demonstraram desempenho diagnóstico limitado nesse grupo de pacientes^28^.

Nosso estudo identificou uma alta incidência (31,1%) de PE sobreposta entre as mulheres com DG. Essa incidência esteve associada a um aumento significativo (p = 0,001) na perda da TFG um ano após a gestação. Estudos sobre desfechos renais de longo prazo não sustentam mais a noção anterior de que a PE é uma condição resolvida com o parto. A PE aumenta o risco de futuras doenças cardiovasculares e renais, sendo o risco de DRC maior em pacientes com doença renal proteinúrica^29^.

### Desfechos Fetais

Evidências crescentes indicam que, mesmo nos estágios iniciais da DRC, o risco de desfechos fetais adversos é maior do que na população em geral^30^. Nosso estudo demonstrou que a gravidez em mulheres com DG representa um alto risco de desfechos fetais adversos, principalmente nos estágios avançados da DRC. Observamos uma taxa geral mais elevada de desfechos fetais adversos: 63,6% (28/44) nos estágios 1 e 2 da DRC, 72,7% (8/11) nos estágios 3a e 3b da DRC e 100% (6/6 gestações) nos estágios 4 e 5.

Observamos uma elevada mortalidade fetal geral (20,4%, 9/44 gestações), que esteve diretamente relacionada ao estágio da DRC. No entanto, demonstrou-se que o uso de hemodiálise intensiva adequada durante a gravidez^11^, quando clinicamente indicado para o controle do estado urêmico, pode levar à redução dessa mortalidade^31^.

Nosso estudo apresenta algumas limitações. Foi realizado em uma única instituição e incluiu um número reduzido de pacientes com diversos tipos de doenças glomerulares. No entanto, é importante observar que todas as pacientes apresentaram doenças glomerulares confirmadas antes do planejamento de suas gestações. Durante a gravidez, elas receberam monitoramento cuidadoso por uma equipe especializada e foram acompanhadas por, pelo menos, um ano após o parto.

Em conclusão, a gravidez é possível para mulheres com DG, mas continua representando um desafio para nefrologistas e obstetras em todo o mundo. Uma gravidez bem-sucedida requer aconselhamento pré-concepcional individualizado, planejamento cuidadoso da gestação e uma abordagem multidisciplinar envolvendo nefrologistas e obstetras especializados em gestações de alto risco.

Nosso estudo indica que a DG aumentou os desfechos adversos materno-fetais e que as importantes alterações na imunidade e na fisiologia renal secundárias à gestação podem atuar como um fator de risco para a progressão da DG. A perda de TFG secundária à gravidez esteve diretamente associada ao estágio da DRC antes da gestação e à PE durante a gravidez. As mulheres que se encontram principalmente nos estágios 4 ou 5 da DRC devem receber aconselhamento pré-concepcional abrangente e individualizado, incluindo uma discussão sobre a possível progressão da DG, a necessidade de diálise durante a gestação e os desfechos materno-fetais.

Embora a gravidez planejada tenha proporcionado melhores desfechos materno-fetais para essas mulheres nos últimos anos, os efeitos da interação entre DG e gravidez vão além do parto. Eles podem afetar a saúde cardiovascular e renal futura tanto das mães quanto de seus bebês, potencialmente prematuros e com baixo peso ao nascer.

## Data Availability

O conjunto completo de dados que fundamenta os resultados desse estudo foi publicado no próprio artigo.
